# Comparable improvements in selective, but not sustained, attention in response to a multi-ingredient nootropic formulation when compared with caffeine

**DOI:** 10.1007/s00394-026-03926-8

**Published:** 2026-03-02

**Authors:** Mark A. Hearris, Carl Langan-Evans, Wee-Lun Foo, Calum Garrigan, Lynn Starke, Catarina Rendeiro, Claire Williams, Philippa Jackson, James P. Morton

**Affiliations:** 1 Institute of Sport, Department of Sports & Exercise Science, Manchester Metropolitan University, Manchester, M1 7EL UK; 2 Research Institute for Sport and Exercise Sciences, Liverpool John Moores University, Liverpool, UK; 3 School of Sport, Exercise & Rehabilitation Sciences, University of Birmingham, Birmingham, UK; 4 School of Psychology & Clinical Language Sciences, University of Reading, Reading, UK; 5 Brain, Performance and Nutrition Research Centre, Faculty of Health and Wellbeing, Northumbria University, Northumbria, UK

**Keywords:** Nootropics, Cognition, Caffeine, Mood

## Abstract

**Purpose:**

The ergogenic effects of caffeine on cognitive performance are largely restricted to improved alertness and enhanced attention and do not typically extend to other cognitive domains. In contrast, plant extracts and phytochemicals exert broader cognitive performance benefits and, when combined together, may provide synergistic effects.

**Methods:**

In a repeated-measures, double-blinded, randomised crossover design, 26 healthy adults completed a cognitive assessment battery 60, 180 and 300 min after ingesting a microcrystalline fibre placebo (PLA), 150 mg caffeine (CAFF) or a multi-ingredient nootropic formulation (NOOT).

**Results:**

Systolic and diastolic blood pressure were significantly higher with CAFF compared to NOOT and PLA (*P* < 0.001). Negative PANAS scores were also significantly higher with CAFF compared to PLA (*P* = 0.039) and NOOT (*P* = 0.033). Arrow flanker correct response reaction times were significantly quicker with CAFF (*P* = 0.009) and NOOT (*P* = 0.012) compared with PLA. Similarly, Stroop correct response reaction times were significantly quicker with CAFF (*P* = 0.017) and NOOT (*P* = 0.039) compared to PLA. RVIP response accuracy was significantly higher with CAFF compared to NOOT and PLA (*P* < 0.001 for both).

**Conclusion:**

Data demonstrate that the multi-ingredient nootropic displayed comparable improvements in selective attention and executive function to caffeine without the negative impact upon blood pressure and subjective mood. However, improvements in sustained attention observed in response to caffeine were not present. Collectively, these data suggest multi-ingredient nootropic formulations have efficacy in improving distinct components of cognitive function, likely dependent on the specific formulation and dose.

**Trial registration number:**

10.17605/OSF.IO/4AB5V (01.08.2025) retrospectively registered.

**Supplementary Information:**

The online version contains supplementary material available at 10.1007/s00394-026-03926-8.

## Introduction

Optimal cognitive functioning is essential across a wide variety of populations, from students and professionals with cognitively demanding careers through to e-gamers and elite athletes who are required to repeatedly process high amounts of visual and perceptual information under intensely demanding conditions. Upon this basis, the use of both pharmacological and/or naturally derived compounds, commonly referred to as nootropics, to enhance cognitive function and subjective mood has received growing interest amongst such populations. This interest is reflected in large-scale global surveys, which highlight the prevalent use of cognitive enhancers amongst both students [1] and medical surgeons [2] who face intense cognitive demands and the need to sustain concentration for prolonged periods. Preliminary survey data also suggests that cognitive enhancers are used in competitive environments, including recreational endurance sports [3] and e-gaming [4] where maintaining attention, decision making and information processing may provide a performance advantage.

Amongst the extensive availability of cognitive enhancers, caffeine is widely recognised for its modulatory effects on the central nervous system which are generally attributed to its role as an adenosine antagonist [5]. As such, caffeine can enhance concentrations of important neurotransmitters such as dopamine, the result of which prevents alertness and attention decrements associated with low arousal [6]. The effects of caffeine on cognitive performance are, however, largely restricted to improved alertness and enhanced concentration or attention and do not typically extend to other cognitive domains such as working memory and executive function [7]. Furthermore, caffeine is also known to increase both systolic and diastolic blood pressure [8] and can often result in increased tension and symptoms of anxiety, nervousness and jitteriness, resulting in deleterious effects on cognition, particularly at higher doses. Therefore, there is growing commercial interest and consumer use of different plant extracts and phytochemicals which are reported to exert broader cognitive performance benefits beyond those of caffeine without the negative side-effects on blood pressure and aspects of subjective mood [9].

In relation to brain function, the use of multi-ingredient formulations that contain a variety of plant extracts and encompass phenolic and terpene groups may provide additive or synergistic effects given their ability to modulate diverse pathways that underpin brain activity. Indeed, data from human intervention studies suggests that phenolic compounds, such as those found in cocoa that are particularly rich in the flavanols catechin and (-)-epicatechin, are able to acutely enhance endothelial function and cerebral blood flow via the production of nitric oxide [10] resulting in improvements in executive function under conditions of high cognitive demand [11]. In addition to the aforementioned phenolic compounds, terpenoids such as those contained within sage species *(Salvia lavandulifolia/officinalis)*, ginkgo (*Ginkgo biloba)*, ginseng (*Panax ginseng)* and bacopa (*Bacopa monnieri*) are also known to modulate neurotransmission via inhibition of enzymes that catalyse the oxidation or hydrolysis of neurotransmitters [12, 13]. For example, the cholinesterase inhibiting properties of sage are thought to modulate cognitive function by inhibiting the hydrolysis of the neurotransmitter acetylcholine [14], with acute doses of 300 to 600 mg being reported to enhance both memory and attention task performance [15]. The properties of sage may become particularly important when co-ingested with citicoline (an acetylcholine precursor) which has been reported to independently augment visual processing speed, working memory and executive function in low baseline performers [16]. Similarly, the main active terpene group within gingko (ginkgolides and bilobalides) can act as GABAA receptor agonists and modulate brain activity by increasing neuronal excitability [17], resulting in improved memory following a single dose [18, 19]. Moreover, the active terpene constituents of ginseng (ginsenosides) and bacopa (bacosides) are structurally similar and, whilst their mechanisms of action are relatively unknown, have been demonstrated to modulate various aspects of cognitive function including memory, attention and executive function [20–23]. Finally, specific amino acids such as taurine, tyrosine and L-theanine have also shown early promise in modulating various aspects of cognition such as choice reaction time [24], working memory [25] and information processing [26], respectively.

Despite the documented effects of each of these compounds on cognitive function in isolation, their potential to provide additive or synergistic effects when combined in a multi-ingredient formulation warrants further investigation given their modulatory effects on the diverse pathways that underpin brain activity. Indeed, assessing the efficacy of a multi-ingredient formulation forms the novel aspect of the present study and reflects growing commercial and consumer interest in such formulations. Accordingly, the primary aim of the present study was to examine the effects of a multi-ingredient formulation containing *Panex ginseng*, *Ginkgo biloba*, sage (Salvia Officinalis L), citicoline, cocoa flavanols, *Bacopa monnieri*, Lion’s mane extract, taurine, tyrosine and L-theanine on subjective mood, physiological responses and cognitive function when compared with caffeine and a negative placebo control. In accordance with the vasodilatory properties of phenolic compounds [27] and potential calming effects of l-theanine [28, 29], we hypothesized that the multi-ingredient nootropic formulation would exert comparable improvements in cognitive function to caffeine without the negative effects on blood pressure and subjective negative mood states.

## Methodology

### Ethical approval

All participants were fully informed of the experimental procedures and potential risks associated with the study before providing written informed consent prior to participation. All trials were conducted at Liverpool John Moores University (Liverpool, UK) following approval from LJMU Research Ethics Committee (UREC) (Approval Ref: 20/SPS/058) in accordance with the latest revision of the Declaration of Helsinki. The study was retrospectively registered within the Open Science Framework (OSF) registry (DOI: 10.17605/OSF.IO/4AB5V).

### Participants

Twenty-six healthy young adults (13 male, 13 female) volunteered to participate in the study. Exclusion criteria for participation included individuals who were current smokers, presented with sleep disturbances or were taking sleep aid medication, had a history of neurological, vascular or psychiatric illness, a current diagnosis of anxiety or depression, a recent history (within 12 months) of alcohol or substance abuse or known food allergies or intolerances to the supplementation or dietary provision. Sample size was calculated a priori and utilised previous assessments of cognitive function in response to multi-ingredient formulations [30]. Based on these data that demonstrate a medium effect size (Cohen’s f = 0.25), a sample size of 26 participants was deemed sufficient to provide statistical power > 0.8 and an α-level of 0.05 (G*Power, Version 3.1, Düsseldorf, Germany).

### Experimental overview

**Fig. 1:**
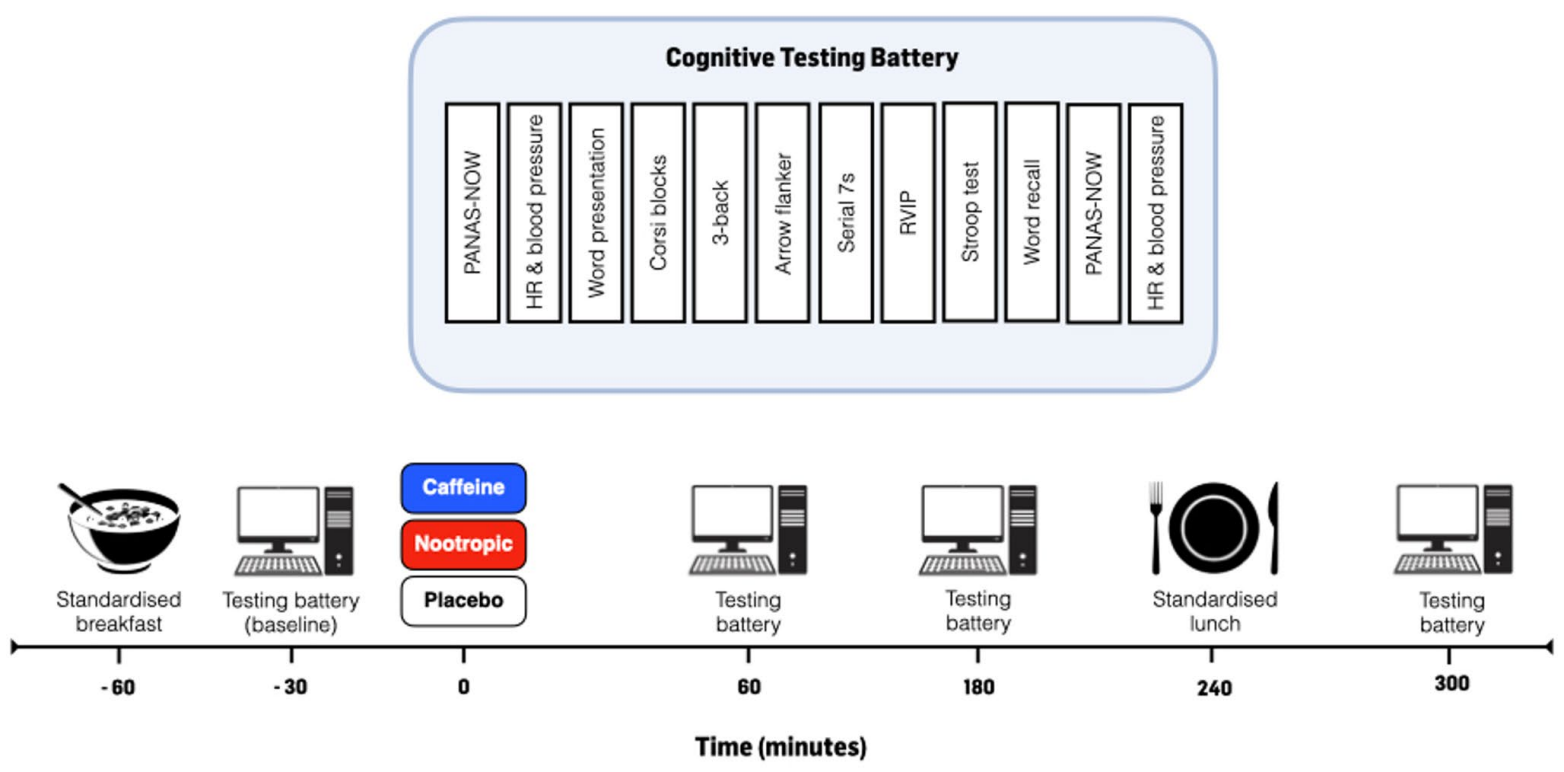
Overview of the experimental protocol

In a repeated-measures, double-blinded, randomised crossover design, participants completed a computerised cognitive assessment battery (COMPASS, Northumbria University, UK) across a 5 h assessment period following the consumption of either placebo (PLA), 150 mg caffeine (CAFF) or a multi-ingredient nootropic formulation (NOOT). Participants reported to the laboratory at ~ 08:00 in the overnight fasted state and were provided with a standardised, low-flavonoid porridge breakfast (described below). Following breakfast, participants completed the computerised cognitive assessment testing battery (baseline) lasting approximately 30 min before consuming one of three experimental treatments (described below). The cognitive assessment battery was further completed at 60, 180 and 300 min following the ingestion of the experimental treatment. A standardised low-flavonoid lunch (described below) was also provided between the third and fourth cognitive testing battery. An overview of the experimental design is presented in Fig. 1.

### Cognitive testing battery

Assessments of cognitive function were administered to participants using the Computerised Mental Performance Assessment System (COMPASS, Northumbria University, Newcastle upon Tyne, UK). The selection of tasks employed as part of the testing battery were chosen to assess various cognitive functions including working memory, selective and sustained attention and executive function which have been shown to be sensitive to various nutritional compounds [31–33]. Participants were fully familiarised with all cognitive assessment tasks on a separate day prior to the completion of the main experimental trials and completed a shortened practice version of each task immediately prior to the ‘true’ assessment task to further minimise any potential learning effects. An overview of each task is presented below.

### Word presentation and delayed word recall (Episodic Memory)

Fifteen words were presented sequentially on screen for one second for participants to remember. During the recognition task, the original fifteen words, plus fifteen distractor words, were presented one at a time in a random order. Participants were required to respond to each word with either “yes” or “no”, indicating whether they were originally presented or not [34]. The presentation of these words and the subsequent delayed word recall task were separated by intervening tasks (Corsi Blocks, 3-Back, Arrow Flanker, Serial 7’s, RVIP, Stroop) resulting in a delay of approximately 20 min between presentation and recall tasks. Outcome measures for this task were accuracy (% correct) and reaction time (mean correct response time in milliseconds).

### Corsi blocks (working memory)

Nine identical blue squares appeared on screen in non-overlapping random positions, with a set number of squares changing colour from blue to red in a randomly generated sequence. Participants were instructed to repeat the sequence by clicking on the blocks with the mouse and cursor. The task was repeated five times at each level of difficulty, increasing from a sequence length of 4 until the participant could no longer correctly recall the sequence [35]. Span score was derived by calculating the mean of the last three correct trials (e.g. 4, 5, 5 = 4.6).

### 3-back (working memory)

A continuous string of letters (upper and lower case) was presented in the centre of the screen for 5 min. For each stimulus, subjects were instructed to indicate whether this was the same letter that appeared three letters before or not by pressing ‘yes’ and ‘no’ buttons as rapidly as possible. A third of all stimuli represented target pairs [36]. Outcome measures for this task were accuracy (% correct), reaction time (mean correct response time in milliseconds) and false alarms (number of incorrect responses).

### Arrow flanker (inhibitory control/selective attention)

Five symbols appeared on the screen with the centre symbol always being an arrow pointing to the left or right. The participant pressed the left or right response buttons corresponding to the direction of the central arrow. The flanking pairs of symbols were either congruent arrows (pointing in the same direction), incongruent arrows (pointing in the opposite direction, or squares (neutral stimuli) [37]. Outcome measures were accuracy (% correct) and reaction time (mean correct response time in milliseconds).

### Rapid visual information processing (RVIP) (sustained attention/vigilance)

The RVIP task required the participant to monitor a continuous series of single digits for targets of three consecutive odd or even numbers. Digits were presented on the computer screen at the rate of 100 per minute, with eight correct target strings in each minute presented in pseudo-random order. Participants responded to the detection of a target string by pressing the appropriate response button as quickly as possible [38]. Outcome measures were accuracy (% correct), reaction time (mean correct response time in milliseconds) and false alarms (number of incorrect responses).

### Serial 7’s (executive function)

The serial 7s task required participants to count backwards in multiples of 7 as quickly and accurately as possible from a randomly generated starting number between 800 and 999, using the keyboards linear number keys to enter each response. Participants were instructed that if they were to make a mistake, they should carry on subtracting from the new incorrect number with subsequent responses scored as correct in relation to the new number [39]. This task was scored for accuracy (% correct).

### Stroop test (inhibitory control/selective attention)

Words describing one of four colours (‘RED’, ‘YELLOW’, ‘GREEN’, and ‘BLUE’) were presented in different coloured fonts in the centre of the computer screen. The participant pressed one of four coloured response buttons in order to identify the font colour (e.g. if the word ‘GREEN’ was presented in a blue font, the correct response would be to respond with the blue button). The presented words were either ‘congruent’ (word and font were the same colour) or ‘incongruent’ (word and font were different colours) and were presented in a random order [40]. Outcome measures were accuracy (% correct) and reaction time (mean correct response time in milliseconds).

### Assessment of subjective mood & physiological responses

Subjective mood scores were measured at the beginning and end of each cognitive testing battery using the Positive and Negative Affect Schedule (PANAS-NOW) which requires participants to rate 20-mood related adjectives, indicating the extent to which they are currently experiencing that emotion on a 5-point Likert scale (1 = not at all, 5 = very much). Half the presented words relate to positive emotions whilst the other half relate to negative emotions. Separate scores were obtained for positive and negative affect by the summation of ratings, with each scale having a maximum score of 50. Resting heart rate and systolic and diastolic blood pressure were measured immediately before and after the completion of each cognitive assessment battery in a seated, upright position (Carescape V100, GE Healthcare, Chicago, US).

### Treatments

Following the completion of the baseline cognitive testing battery, participants consumed one of three nutritional supplements in a randomised, counterbalanced order. All supplements were provided in 6 visually identical opaque capsules and contained either microcrystalline fibre (PLA), 150 mg caffeine (CAFF) or a multi-ingredient nootropic formulation (NOOT) containing 400 mg *Panex ginseng*, 360 mg *Ginkgo biloba*, 300 mg sage (Salvia Officinalis L), 500 mg citicoline, 500 mg cocoa flavanols, 320 mg *Bacopa monnieri*, 500 mg Lion’s mane extract, 250 mg taurine, 500 mg tyrosine and 250 mg L-theanine. Independent external analysis of each ingredient by the ingredient supplier revealed that ginkgo contained 24.4% ginkgo flavone glycosides and 6.3% total terpene lactones, ginseng contained 4.5% total ginsenosides and bacopa contained 51.1% bacosides. Concentrations of ginkgo flavone glycosides, terpene lactones and bacosides were determined by high performance liquid chromatography (HPLC) whilst total ginsenoside content was determined using the UV calorimetric method.

### Nutritionali intake

In the 24 h prior to each experimental visit, participants were asked to refrain from caffeine, alcohol and any form of structured physical exercise. Participants were also provided with a list of high-flavonoid foods to avoid during this period. To ensure adherence to these instructions and allow for replication in subsequent experimental trials, participants were instructed to record all food and drink intake during this period. Adherence to these instructions was evaluated using the Remote Food Photography Method, where participants provided images of all food and drink consumed during this period. Whilst we acknowledge that 24 h restriction of high-flavonoid foods may not eliminate all circulating bioactive metabolites, our primary intention was to minimise the inter- and intra-subject variability in bioactive metabolites whilst still maintaining an appropriate level of ecological validity. Upon arrival to the laboratory, participants were provided with a standardised, low-flavonoid porridge breakfast containing 70 g carbohydrate (CHO), 16 g protein and 6 g fat. For all participants, breakfast was consumed 30 min prior to the start of baseline tests and 60 min prior to the ingestion of the nutritional supplement. A standardised low-flavonoid lunch containing a sandwich, 1 × 25 g ready salted crisps and a banana containing 110 g CHO, 16 g protein and 22 g fat was consumed between the third and fourth cognitive testing battery.

### Statistical analysis

All statistical analyses were performed using SPSS Statistics Version 29 (IBM, US). A linear mixed model, with random intercepts to account for repeated measurements within subjects and baseline data as the covariate, was used to analyse differences over time and between treatments for all outcome measures. Where a significant main effect was observed, pairwise comparisons were analysed using post-hoc LSD tests to locate specific differences. The use of LSD post-hoc tests were selected as multiplicity adjustment is not required when evaluating distinct interventions, whereby doing so may increase type II error. This approach aligns with recommendations that individual treatment effects are the primary focus in multi-arm trials [41]. All data in text, figures and tables are presented as estimated marginal means ± SEM with *P* values ≤ 0.05 indicating statistical significance.

## Results

### Physiological responses

**Fig. 2:**
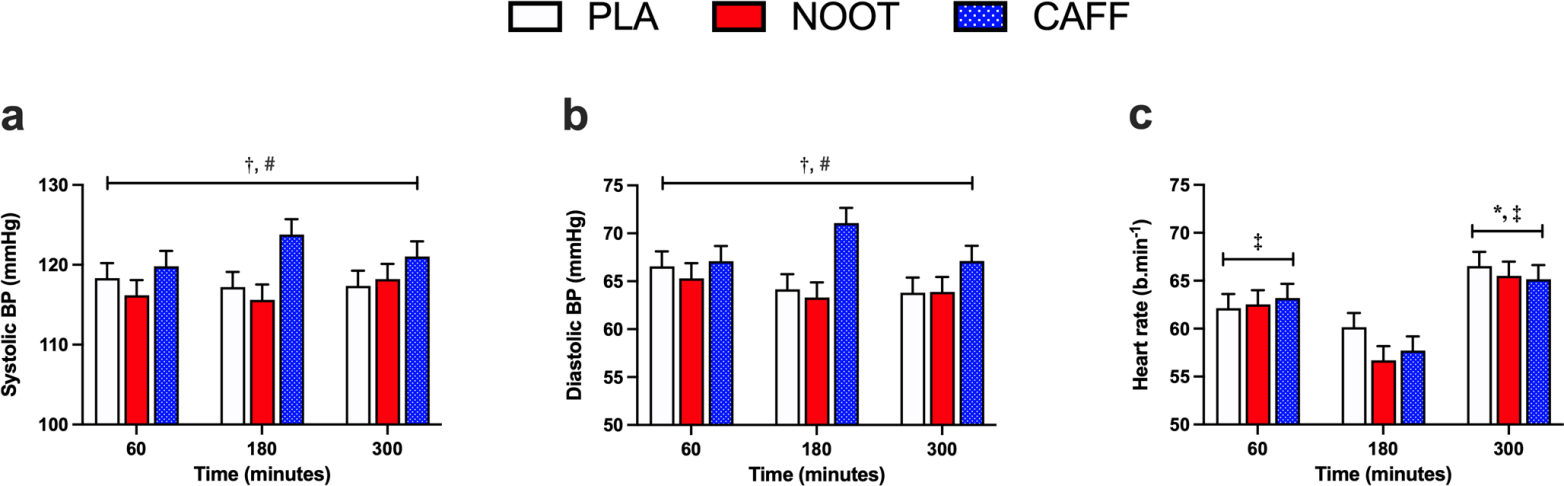
Systolic **a** and diastolic **b** blood pressure and **c** resting heart rate at 60, 180 and 300 min following the ingestion of either placebo (PLA), multi-ingredient nootropic (NOOT) or caffeine (CAFF). **†** significant difference between placebo and caffeine, **#** significant difference between nootropics and caffeine, **‡** significantly different to 180 min, * significantly different to 60 min. *P* < 0.05

Both systolic and diastolic blood pressure were significantly higher in response to CAFF (treatment effect, *P* < 0.001 for both) when compared with both NOOT and PLA trials (*P* < 0.001 for all comparisons) across the treatment period (Fig. 2). Whilst systolic blood pressure did not change over time (time effect, *P* = 0.657), diastolic blood pressure was significantly higher (time effect, *P* = 0.013) at 60 when compared with 300 min (*P* = 0.004). No significant differences in resting heart rate (treatment effect, *P* = 0.221) were observed between conditions, however, heart rate was significantly higher (time effect, *P* < 0.001) at 300 min when compared with both 60 and 180 min (*P* < 0.001 for both). Resting heart rate was also higher at 60 min when compared with 180 min (*P* < 0.001).

### Subjective mood

**Fig. 3:**
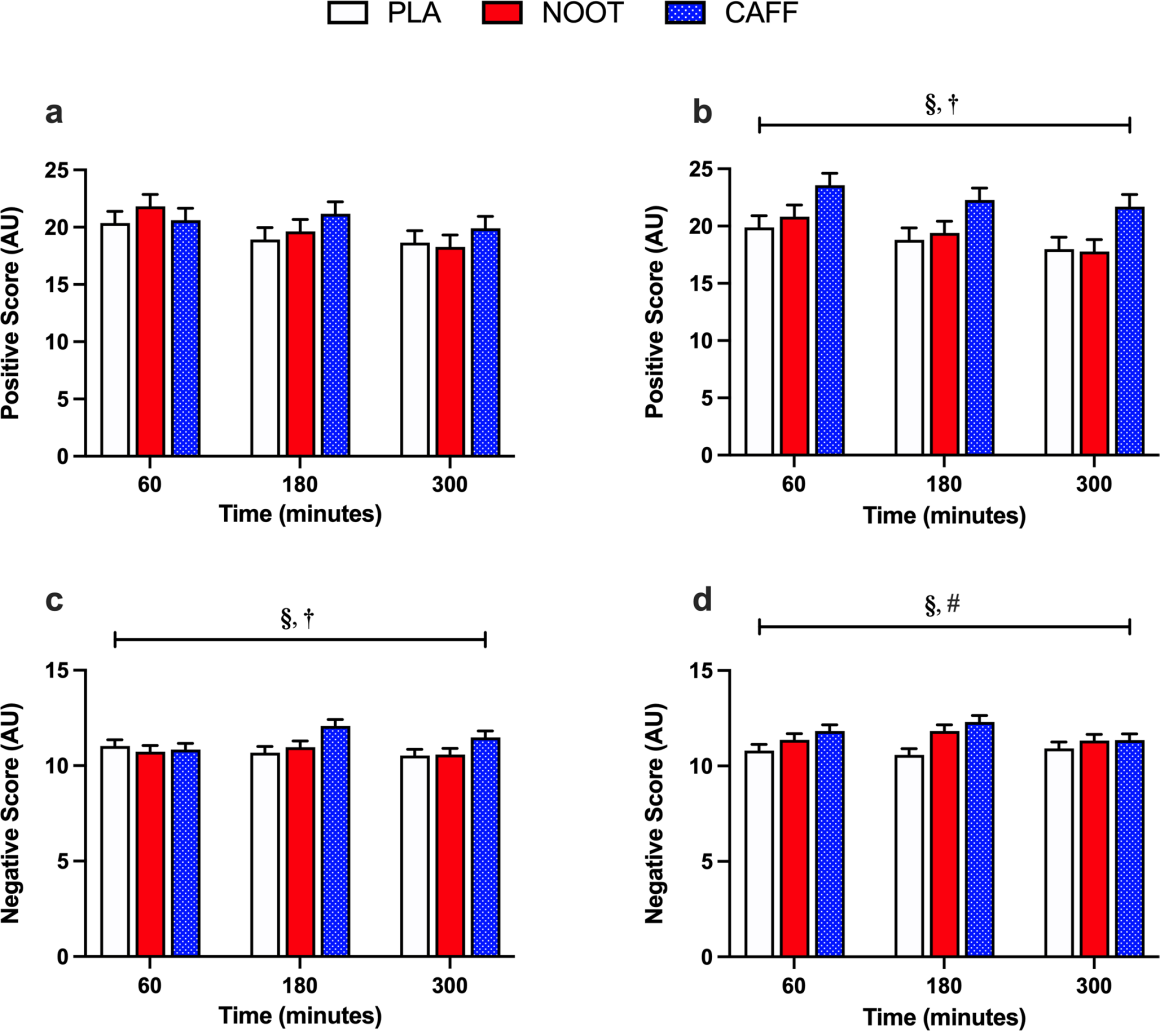
Positive (**a**,**b**) and negative (c-d) PANAS-NOW scores before (**a**,**c**) and after (**b**,**d**) completing the cognitive assessment battery following the ingestion of either placebo (PLA), multi-ingredient nootropic (NOOT) or caffeine (CAFF). **§** significant difference between CAFF and PLA, **†** significant difference between CAFF and NOOT, **#** significant difference between PLA and NOOT. *P* < 0.05

Positive PANAS scores were significantly higher (treatment effect, *P* = 0.004) in response to CAFF when compared with both NOOT and PLA trials (*P* < 0.001 for both) (Fig. 3). Importantly, these differences were only observed following the completion of each testing battery with no differences present prior to each testing battery (treatment x period interaction, *P* < 0.001). Negative PANAS scores were also significantly higher (treatment effect, *P* = 0.004; treatment x period interaction, *P* = 0.014) in response to CAFF when compared with PLA (*P* = 0.039) and NOOT (*P* = 0.033) prior to each testing battery. Furthermore, negative PANAS scores remained significantly higher in response to CAFF when compared with PLA (*P* = 0.001) following the completion of each testing battery. Whilst there were no differences in negative PANAS scores between NOOT and PLA trials prior to each testing battery (*P* = 1.000), negative scores were significantly higher in the NOOT trial following the completion of the testing battery (*P* = 0.022).

### Cognitive responses

#### Arrow flanker

**Fig. 4:**
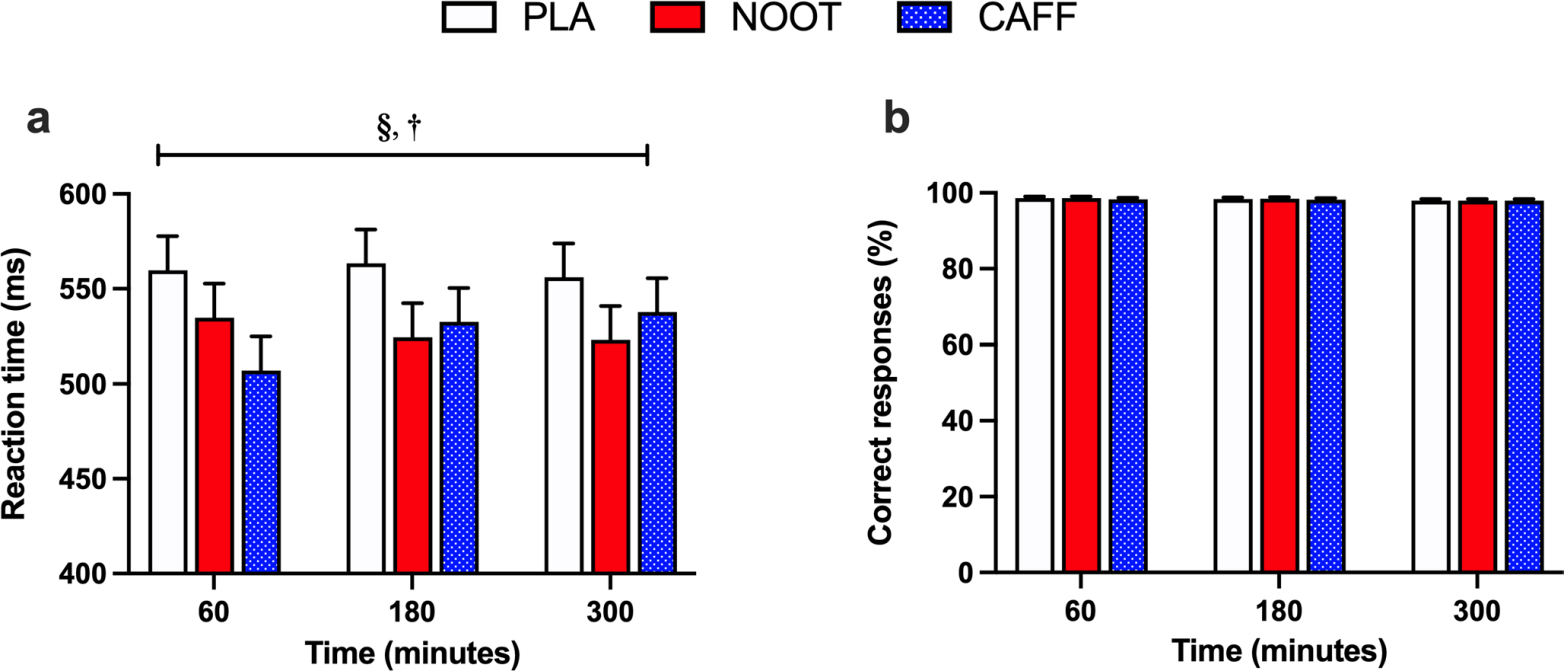
Correct response reaction time (**a**) and response accuracy (**b**) during the arrow flanker test at 60, 180 and 300 min following the ingestion of either placebo (PLA), multi-ingredient nootropic (NOOT) or caffeine (CAFF). **§** significant difference between placebo and nootropics, **†** significant difference between placebo and caffeine, *P* < 0.05

Correct response reaction times during the arrow flanker test were significantly quicker (treatment effect, *P* = 0.004) in response to both CAFF (*P* = 0.003) and NOOT (*P* = 0.004) trials when compared with PLA, respectively across the 300 min study period (Fig. 4). Response accuracy (i.e. number of correct responses) was not significantly different between conditions (treatment effect, *P* = 0.695). Furthermore, neither correct response reaction times (time effect, *P* = 0.803) or response accuracy (time effect, *P* = 0.062) was significantly different between timepoints.

#### 3-Back

In response to the 3-back test, no significant differences in accuracy (treatment effect, *P* = 0.546), correct response reaction time (treatment effect, *P* = 0.434) or error rate (treatment effect, *P* = 0.728) were observed between conditions. 3-back accuracy was significantly higher (time effect, *P* = 0.003) at 180 min when compared with both 60 (*P* = 0.004) and 300 (*P* = 0.003) minute timepoints. In contrast, neither correct response reaction time (time effect, *P* = 0.054) or error rate (*P* = 0.565) was significantly different between timepoints.

#### Stroop

**Fig. 5:**
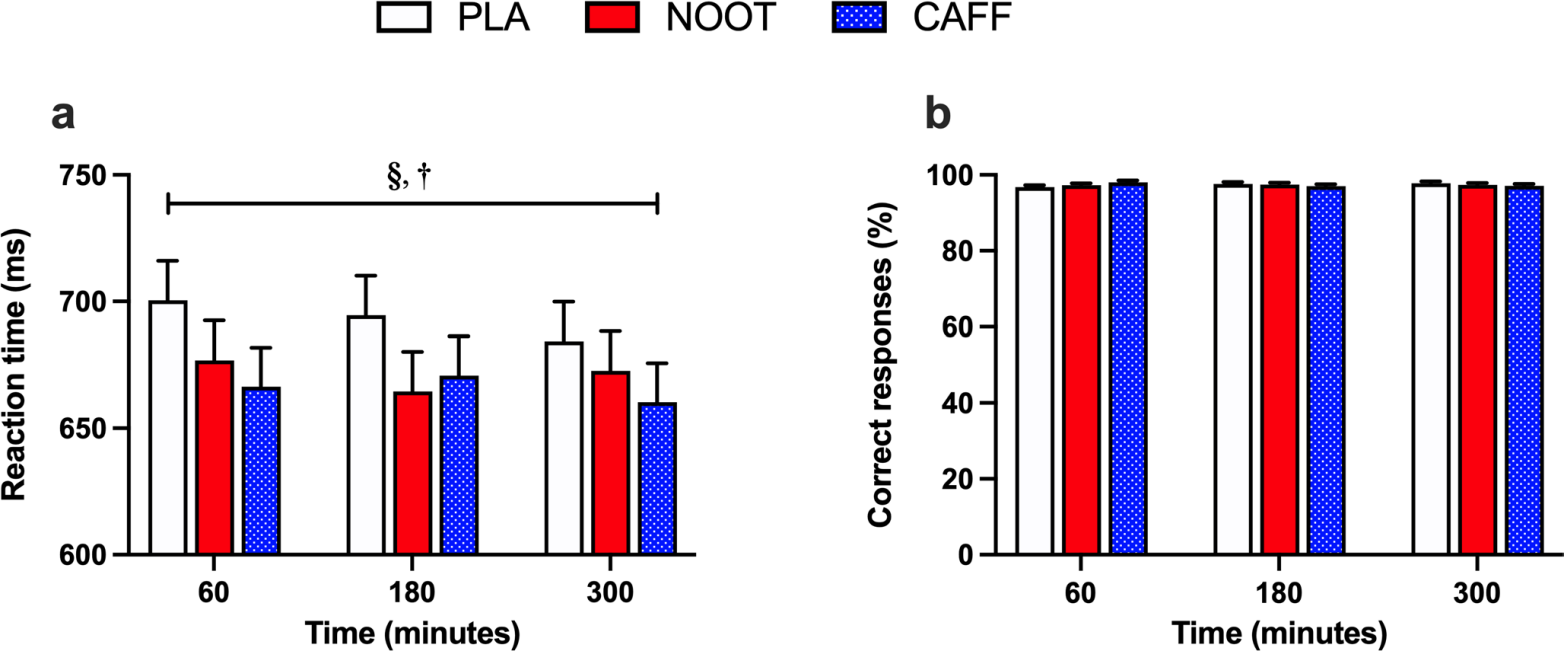
Correct response reaction time (**a**) and response accuracy (**b**) during the Stroop test at 60, 180 and 300 min following the ingestion of either placebo (PLA), multi-ingredient nootropic (NOOT) or caffeine (CAFF). **§** significant difference between placebo and nootropics, **†** significant difference between placebo and caffeine, *P* < 0.05

Correct response reaction times during the Stroop test were significantly quicker (treatment effect, *P* = 0.036) in response to both CAFF (*P* = 0.017) and NOOT (*P* = 0.039) trials when compared with PLA across the 300 min study period (Fig. 5). Response accuracy (i.e. number of correct responses) was not significantly different between conditions (treatment effect, *P* = 1.000). Furthermore, neither correct response reaction times (time effect, *P* = 0.512) or accuracy (time effect, *P* = 0.976) were significantly different between timepoints.

### Rapid visual information processing

**Fig. 6:**
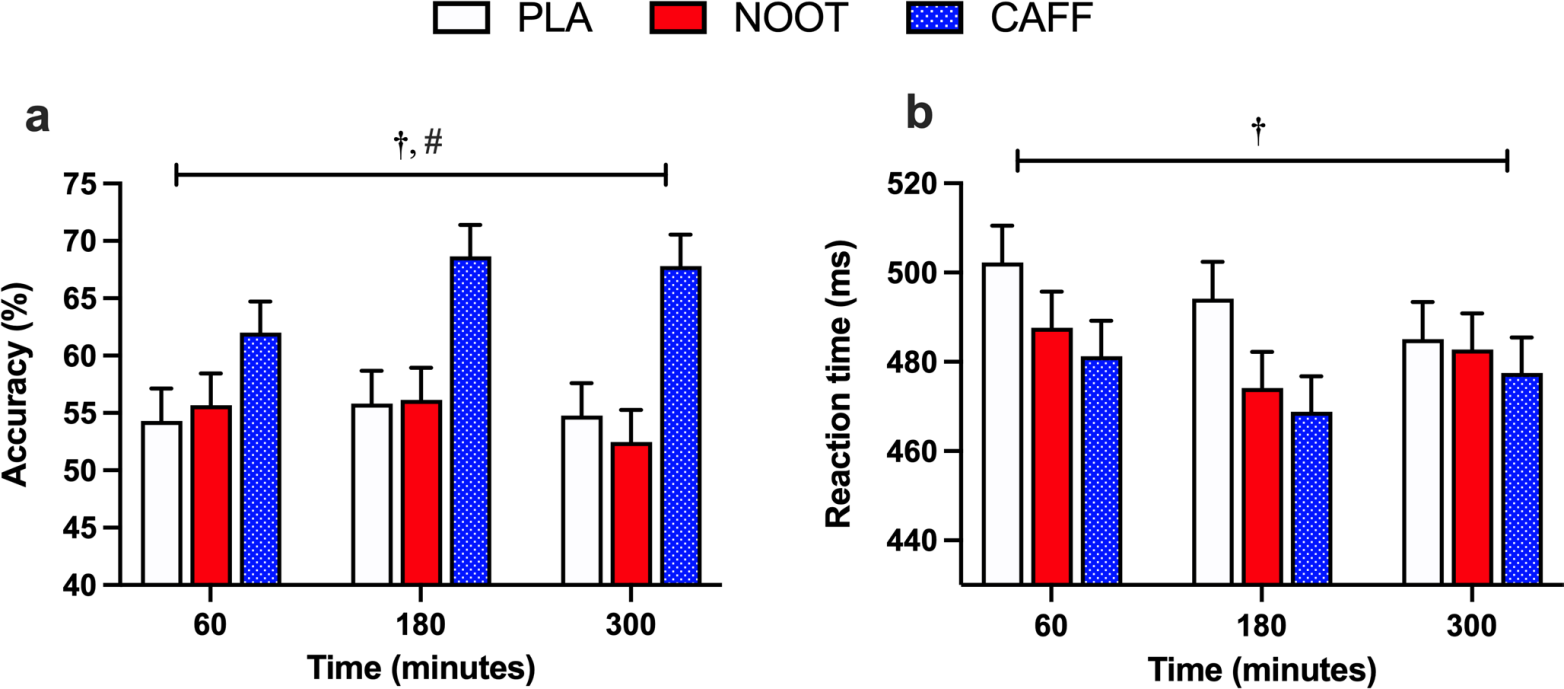
Response accuracy (**a**) and reaction time (**b**) during the RVIP test at 60, 180 and 300 min following the ingestion of either placebo (PLA), multi-ingredient nootropic (NOOT) or caffeine (CAFF). **†** significant difference between placebo and caffeine, **#** significant difference between nootropics and caffeine. *P* < 0.05

Response accuracy during the RVIP test was significantly higher (treatment effect, *P* < 0.001) in response to the CAFF trial when compared with both NOOT and PLA trials, respectively (*P* < 0.001 for both) with no differences in accuracy between NOOT and PLA trials (*P* = 1.000). Correct response reaction times were also significantly quicker (treatment effect, *P* = 0.031) in response to the CAFF trial, but only when compared with PLA (*P* = 0.011) with no differences in reaction times between NOOT and PLA trials (*P* = 0.391) (Fig. 6). Furthermore, neither response accuracy (time effect, *P* = 0.180) or correct response reaction times (time effect, *P* = 0.132) were significantly different between timepoints.

### Serial 7s

**Fig. 7:**
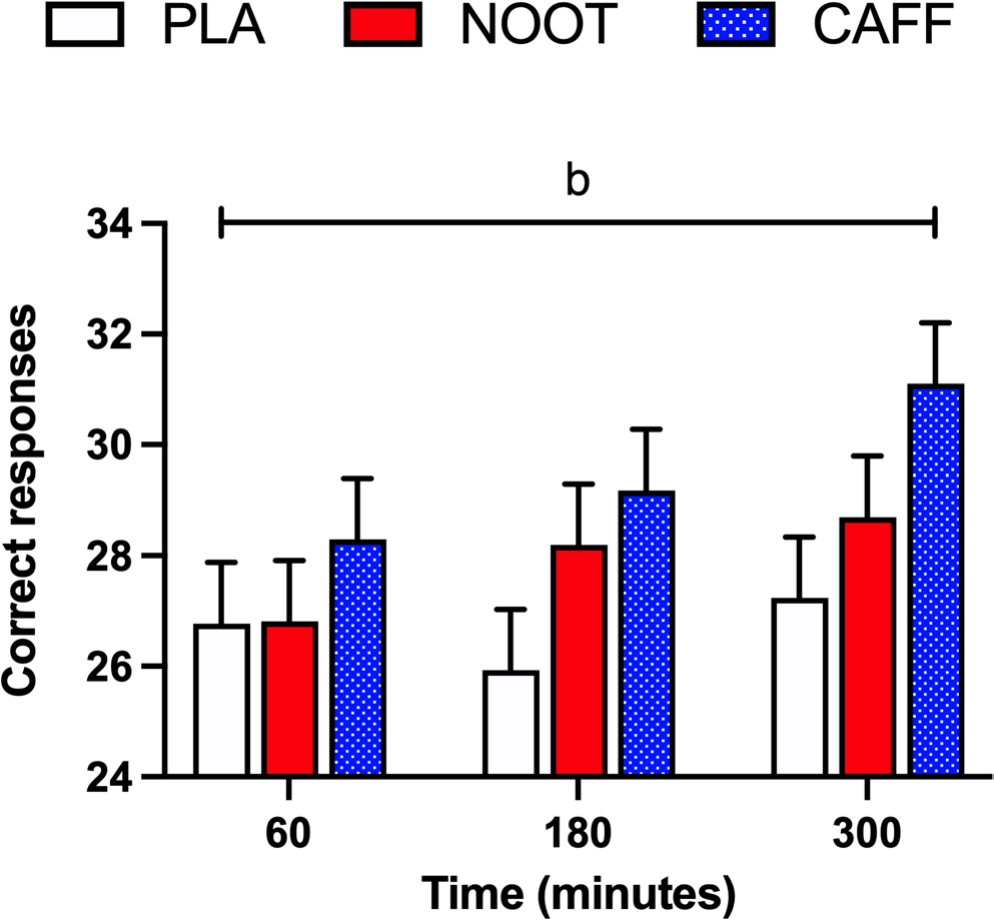
Correct responses during the Serial 7’s test at 60, 180 and 300 min following the ingestion of either placebo (PLA), multi-ingredient nootropic (NOOT) or caffeine (CAFF). **†** significant difference between placebo and caffeine. *P* < 0.05

The number of correct responses given during the Serial 7s test was significantly higher (treatment effect, *P* = 0.019) in response to the CAFF trial when compared with PLA (*P* = 0.006) with no differences between CAFF and NOOT trials (*P* = 0.092) (Fig. 7). In contrast, the error rate was not significantly different between conditions (treatment effect, *P* = 0.233). Neither the number correct responses (time effect, *P* = 0.076) or the error rate (time effect, *P* = 0.248) were significantly different between timepoints.

### Word recognition

No significant differences in word recognition accuracy (treatment effect, *P* = 0.053) or correct response reaction time (treatment effect, *P* = 0.927) were observed between conditions. Furthermore, neither word recognition accuracy (time effect, *P* = 0.620) or correct response reaction time (time effect, *P* = 0.957) were significantly different between timepoints.

### Corsi blocks

No significant differences in span score were observed between conditions (treatment effect, *P* = 0.420) or timepoints (time effect, *P* = 0.185).

## Discussion

Using a well-established battery of cognitively demanding tasks, we examined the effect of a novel multi-ingredient nootropic formulation on cognitive function and subjective mood states across a five-hour period. We provide novel data by demonstrating comparable improvements in arrow flanker and Stroop task performance in response to the nootropic formulation when compared with those observed with caffeine, in the absence of the observed negative effects of caffeine on resting blood pressure and subjective mood. Nonetheless, the expected improvements in performance of the RVIP task observed following caffeine administration, were not present in response to the nootropic formulation, suggesting a unique profile of effects specific to the formulation and dose provided in the current study. Collectively, these data highlight the efficacy of the nootropic formulation during tasks that require selective attention and inhibitory control but not those associated with sustained attention.

In response to the caffeine control, we observed a consistent, yet limited range of cognitive effects in line with previous findings [42, 43]. Specifically, the acute dose of 150 mg caffeine resulted in improvements across tasks assessing attention and vigilance (arrow flankers, Stroop, RVIP) which were sustained over the five hour testing period in accordance with caffeine’s typical 3–5 h half-life [44]. Performance on the Serial 7’s task, which requires both attention and working memory, was also improved following caffeine, adding to the literature of its inconsistent effects on this task [45, 46]. In contrast, both long-term (word recognition) and working (Corsi blocks, 3-back) memory tasks were unaffected by caffeine. Importantly, these data also confirm that the established testing battery was sensitive to detect acute changes in cognitive performance in response to nutritional interventions in accordance with previous studies utilising this approach [31–33]. In response to the multi-ingredient nootropic formulation, we observed comparable improvements in correct response times on both Stroop and arrow flanker tasks which require selective attention and top-down control to ignore distracting stimuli (arrow flankers) or automatic responses (Stroop task) when compared with placebo. However, similarly to caffeine, the enhanced cognitive improvements exerted by the nootropic formulation were limited to a small selection of cognitive tasks and failed to extend to aspects of long-term memory (word recognition) and working memory (corsi blocks, 3-back) tasks. In considering this, our data suggest that a single or small number of the included ingredients may be responsible for the observed outcomes. Whilst it is inherently difficult to ascertain the individual components that underpin the observed effects within the current experimental design, we consider tyrosine, l-theanine and ginseng as potential candidates given their reported cognitive effects.

Previous studies with tyrosine have shown promise in enhancing working memory and executive function in healthy, young adults under conditions of high stress when adopting doses of 2 g or higher [47]. In particular, similar improvements in Stroop task response times to those observed in the present study have been demonstrated following an acute dose of 2 g [48]. In addition to tyrosine, l-theanine has been suggested to decrease neural resource allocation to process distractors thereby enhancing the ability to concentrate on target stimuli more effectively and may help explain the observed improvements in Stroop task response times. Whilst previous data suggest that l-theanine, in the absence of caffeine, has minimal effects of cognition [7], acute doses of 100 mg have been demonstrated to enhance Stroop task response time, albeit in healthy older adults [49]. Furthermore, acute doses of 200 mg administered to healthy males appear to reduce fMRI activity in brain areas regulating visual attention during a colour discrimination task, supporting the notion that l-theanine may enhance the ability to concentrate on target stimuli [50]. Improvements in attentional task performance have also been observed following acute doses of ginseng amongst healthy, young adults. For example, acute doses of 200–400 mg of ginseng have been shown to enhance speed of attention task performance up to 6 h post-dose [51] whilst doses of 400 mg demonstrate improved accuracy of attention tasks up to 2.5 h post-dose [21]. Interestingly, reductions in P300 latency, indicative of faster cognitive processing, have been observed following an acute dose of 200 mg ginseng (G115) extract and may help to explain the reported improvements in speed of task performance [52].

Whilst positive subjective affect scores were higher in response to caffeine across the intervention period, this effect was only present following, rather than prior to, the completion of the cognitive testing battery. This effect is likely explained by the well-established alerting effects of caffeine, attenuating the negative effects of the highly demanding testing battery. Although previous research has demonstrated increased feelings of vigour, arousal and alertness following comparable doses (150 mg) of caffeine prior to the completion of cognitive assessment tasks [53–55], effects on specific emotions may have been masked when summarised alongside the wide-range of positive emotions captured with the PANAS-NOW in the present study. Interestingly, negative affect scores were also increased in response to caffeine across the intervention period and persisted following the cognitive testing battery despite the concomitant increase in subjective positive affect scores observed at this time. These results are likely driven by the known negative effects of caffeine, including increased anxiety and jitteriness which have been previously observed in response to comparable doses [53–55]. Whilst we also observed similar increases in negative affect scores following the testing battery with the nootropic formulation, it is difficult to ascertain whether these effects are isolated to individual ingredients or occurred as a result of unknown interactions between ingredients. For instance, cocoa flavanols [56], ginseng [21], ginkgo biloba [21], sage [15] and bacopa monnieri [20] have all independently been demonstrated to enhance aspects of subjective mood at comparable doses to those within the nootropic formulation. Thus, in considering that none of the nootropic ingredients have been reported to negatively influence subjective mood in isolation, interactions between individual ingredients may explain the present findings.

In considering the present cognitive outcomes, it is unclear why the observed improvements in cognition were limited to those of selective attention/inhibitory control despite the array of ingredients in the nootropic formulation and their expected broad-spectrum of effects. Whilst the answer is likely comprised of several factors, appropriate dosing of multiple ingredients within a single formulation is often practically challenging, especially when delivered in capsule format to ensure appropriate blinding as per the current study design. As in the case of tyrosine, when compared with previous studies that demonstrate positive cognitive effects at doses of 2 g, it is possible that the present dose of 500 mg may be insufficient to exert its beneficial effects. Second, for ingredients such as bacopa [57, 58], citicoline [59] and lion’s mane [60], the literature indicates chronic supplementation may be more appropriate. Finally, in considering that it is not uncommon for combinations of bioactive ingredients to exert a different profile of effects compared to individual administration, it is plausible that some ingredients may have counteracted the effects of others or negatively interacted with one another to impair specific cognitive functions. For example, combined ingestion of ginseng/gingko biloba can enhance accuracy scores across a range of memory tasks yet impair reaction times across a series of attention tasks when compared with individual administration of each ingredient [61]. Nonetheless, despite the potential for counteractions between individual nutrients, we did not observe any reductions in cognitive performance in response to the nootropic formulation when compared with placebo.

From a practical perspective, the inclusion of various bioactive nootropic ingredients may have the most efficacy when combined with caffeine. In accordance with the present data, it is well-established that the effects of caffeine on cognitive performance are largely restricted to improved alertness and enhanced concentration or attention and do not typically extend to other cognitive domains. Furthermore, as evidenced in the present study, caffeine consistently enhances resting blood pressure and is associated with negative emotions such as anxiety and jitteriness. As such, combining caffeine with other bioactive ingredients that exert broader cognitive enhancing effects whilst simultaneously reducing the negative symptoms associated with caffeine may be where multi-ingredient formulations can be most efficacious. For example, combining l-theanine with caffeine has been previously reported to further augment the behavioural and mood effects of caffeine alone [29, 50] whilst attenuating the rise in both systolic and diastolic blood pressure [62]. When considering the efficacy of ingredients for multi-ingredient formulations, it may be more appropriate to combine a small number of additional ingredients to ensure appropriate dosing (avoiding the need for ‘propriety blends’) and minimise the potential for negative or counteractive effects.

Despite the novelty and practical relevance of our data, we acknowledge that our study has several limitations which should be addressed. Firstly, we recognise that the current study design prevents us from isolating the effects of each individual ingredient or their potential interactions and, as such, the observed outcomes may reflect the influence of a single ingredient, a subset of ingredients, or additive effects arising from their combination. Second, although our study was appropriately powered to detect medium effect sizes based on prior work using multi-ingredient formulations [30], some cognitive outcomes may exhibit smaller effects in response to nutritional interventions and thus require larger sample sizes to detect such effects. Finally, whilst the present data demonstrate the cognitive enhancing potential of multi-ingredient formulations, the underlying neurobiological mechanisms remain unclear and require further research.

In conclusion, our data demonstrates that a novel multi-ingredient nootropic formulation can elicit measurable improvements in selective attention and inhibitory control, comparable to those observed with caffeine, but without the accompanying increases in resting blood pressure and negative mood effects. While these cognitive benefits did not extend to working or long-term memory tasks, the findings provide valuable evidence for the targeted use of specific ingredient formulations to enhance aspects of cognitive performance. Although these data collectively underscore the potential efficacy of multi-ingredient nootropic formulations, future work should investigate the efficacy of combining caffeine with other bioactive ingredients that exert broader cognitive enhancing effects whilst simultaneously reducing the negative symptoms associated with caffeine. Such work should also aim to provide further mechanistic insight through the use of neuroimaging techniques as well as the measurement of cerebral blood flow and metabolite pharmacokinetics to extend our understanding of how such ingredients modulate cognitive function outcomes.

## Supplementary Information

Below is the link to the electronic supplementary material.

Supplementary Material 1

## Data Availability

The datasets used and/or analysed during the current study are available at 10.23634/MMU.00641204.
