# Supplementary material for: Comparable improvements in selective, but not sustained, attention in response to a multi-ingredient nootropic formulation when compared with caffeine
Source: Eur J Nutr. 2026 Mar 2;65(2):75. doi: 10.1007/s00394-026-03926-8 (PMC12953322; doi:10.1007/s00394-026-03926-8)
Supplement: Supplementary file 1 — Supplementary Material 1 [file 394_2026_3926_MOESM1_ESM.docx]

| **Outcome measure** | **Treatment** | **Time** | **Period** | **Treatment*Time** | **Treatment*Period** |
| --- | --- | --- | --- | --- | --- |
| **Physiological Outcomes** | | | | | |
| Systolic blood pressure | < 0.001* | 0.013* | 0.737 | 0.219 | 0.965 |
| Diastolic blood pressure | < 0.001* | 0.657 | 0.029* | 0.339 | 0.728 |
| Resting heart rate | 0.221 | < 0.001* | 0.574 | 0.114 | 0.491 |
| **Subjective Mood** | | | | | |
| Positive PANAS | 0.004* | < 0.001* | 0.308 | 0.687 | < 0.001* |
| Negative PANAS | 0.004* | 0.062 | < 0.001* | 0.066 | 0.014* |
| **Cognitive Responses** | | | | | |
| **Arrow Flanker** | | | | | |
| Correct response RT | 0.004* | 0.803 | - | 0.465 | - |
| Response accuracy | 0.695 | 0.062 | - | 0.962 | - |
| **3-Back** | | | | | |
| Accuracy | 0.546 | 0.003* | - | 0.094 | - |
| Correct response RT | 0.434 | 0.054 | - | 0.060 | - |
| Error rate | 0.728 | 0.565 | - | 0.602 | - |
| **Stroop** | | | | | |
| Correct response RT | 0.036* | 0.512 | - | 0.759 | - |
| Response accuracy | 1.000 | 0.976 | - | 0.192 | - |
| **RVIP** | | | | | |
| Response accuracy | < 0.001* | 0.180 | - | 0.358 | - |
| Correct response RT | 0.031* | 0.132 | - | 0.721 | - |
| **Serial 7s** | | | | | |
| Correct responses | 0.019* | 0.076 | - | 0.657 | - |
| Error rate | 0.233 | 0.248 | - | 0.458 | - |
| **Word recognition** | | | | | |
| Accuracy | 0.053 | 0.620 | - | 0.706 | - |
| Correct response RT | 0.927 | 0.957 | - | 0.341 | - |
| **Corsi blocks** | | | | | |
| Span score | 0.420 | 0.185 | - | 0.546 | - |

**Supplementary Table 1.** Statistical outcomes (p values) for all outcome measures. Period refers to the period of measurement (before or after the cognitive testing battery) given that physiological outcomes and subjective mood were measured on two separate occasions (periods) at each time point.

*indicates statistical significance
